# Supplementary material for: Enhanced Site-Specific Fluorescent Labeling of Membrane Proteins Using Native Nanodiscs
Source: Biomolecules. 2025 Feb 10;15(2):254. doi: 10.3390/biom15020254 (PMC11852578; doi:10.3390/biom15020254)
Supplement: Supplementary file 1 [file biomolecules-15-00254-s001.zip › biomolecules-3352674-supplementary.pdf]

Supplementary Information

Enhanced site-specific fluorescent labeling of membrane proteins using native nanodiscs

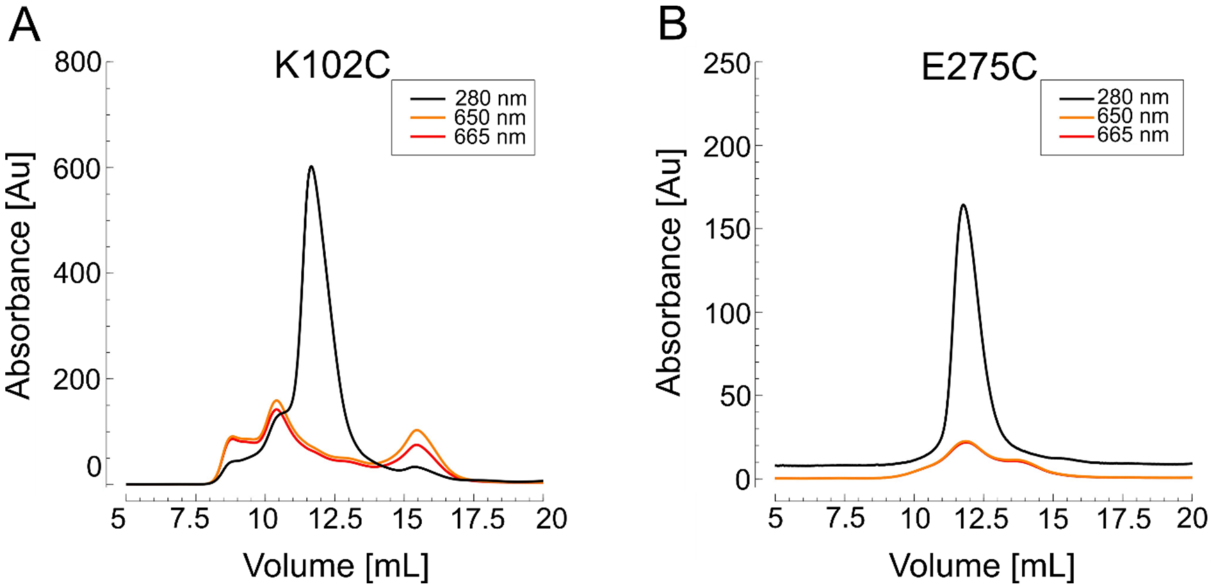

Figure S1. SEC of detergent-solubilized KvAP with point-mutated cysteine residues, labeled with AF647 maleimide. The black curve represents UV absorbance, while the orange and yellow curves indicate fluorescence emission at 650 nm and 665 nm, respectively. The tetrameric protein elutes at 10–12.5 mL.

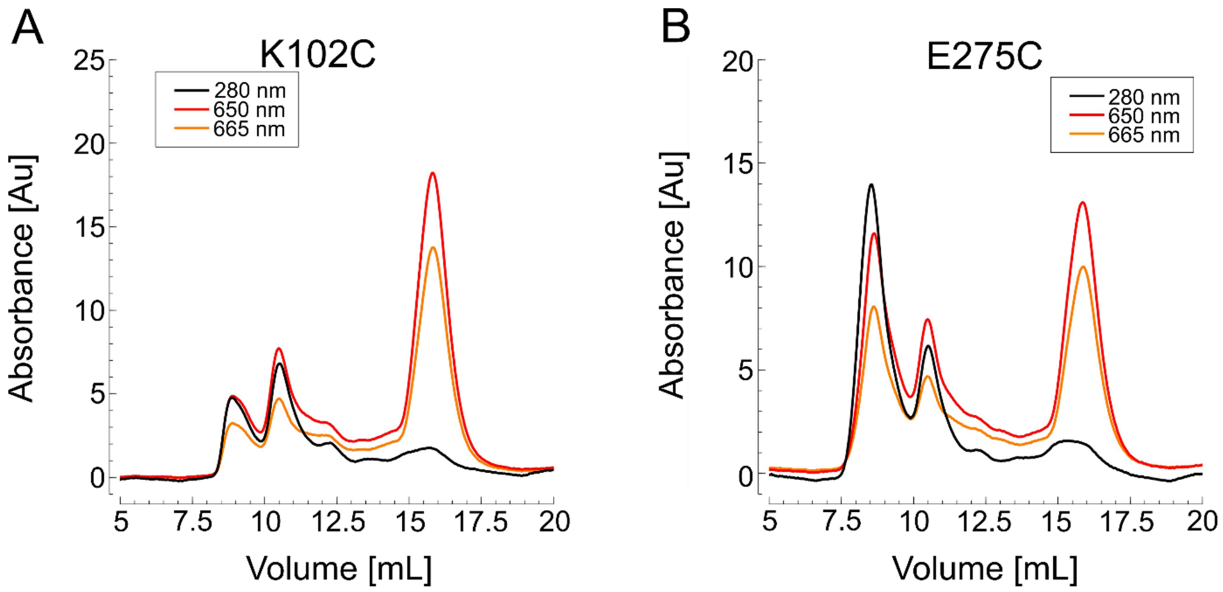

Figure S2. SEC of nanodisc-embedded KvAP with single-cysteine mutations, labeled with AF647 maleimide***.*** The black curve represents UV absorbance, while the orange and yellow curves indicate fluorescence emission at 650 nm and 665 nm, respectively. The tetrameric protein elutes at10–12.5 mL.

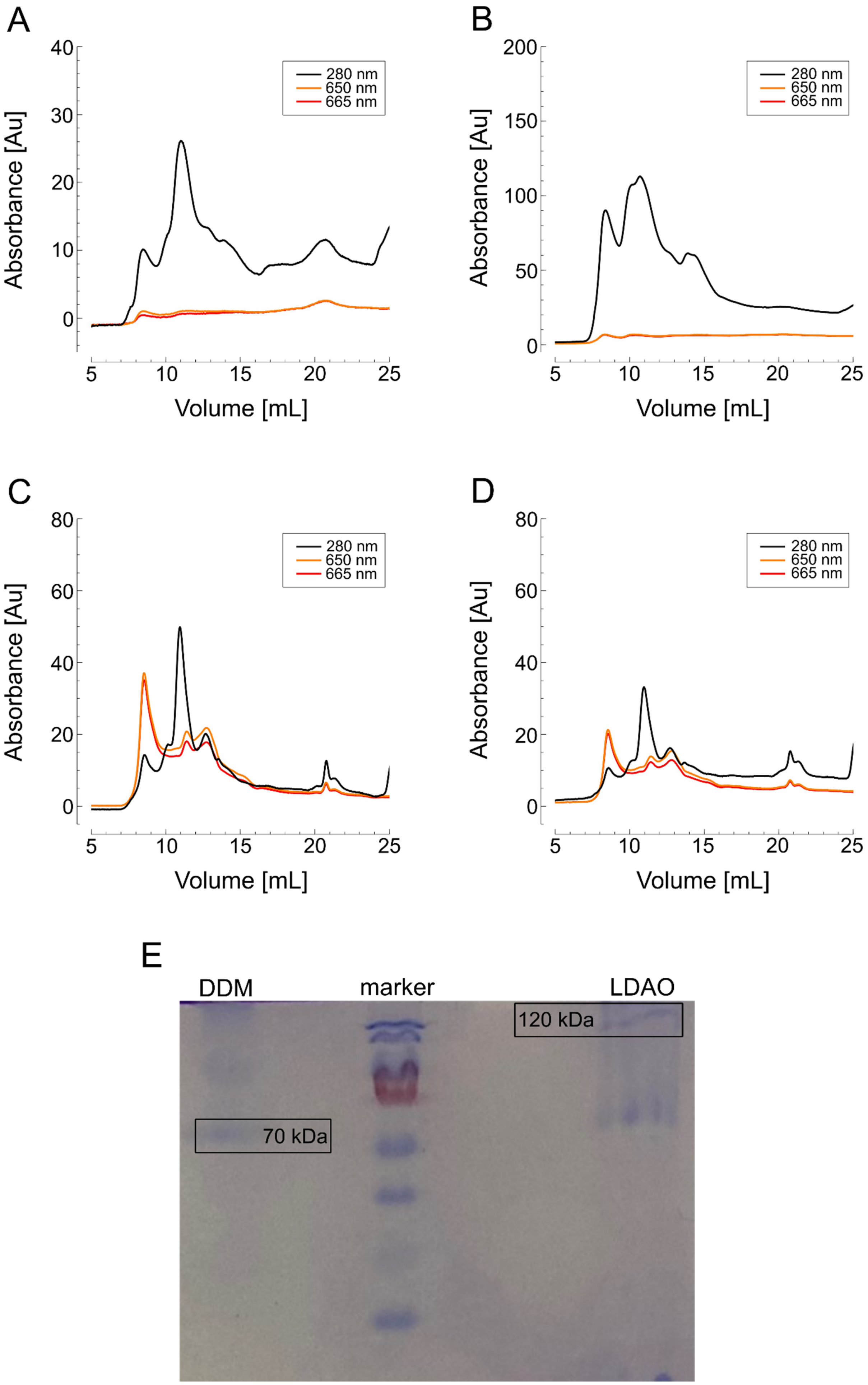
A
B
C
D
E
Absorbance [Au]
Volume [mL]
280 nm
650 nm
665 nm
DDM
marker
LDAO
70 kDa
120 kDa

Figure S3. SEC of HpUreI, purified with DDM or LDAO and labeled with AF647 maleimide. The black curve represents UV absorbance, while the orange and yellow curves indicate fluorescence emission at 650 nm and 665 nm, respectively. *A*: HpUreI purified in DDM, labeled in solution, without TCEP. *B*: HpUreI purified in DDM, labeled on column, without TCEP. *C*: HpUreI purified in DDM, labeled in solution, with TCEP. *D*: HpUreI purified in DDM, labeled on column, with TCEP. *E*: HpUreI purified in LDAO, labeled on column, with TCEP.
